# MicroRNA-mRNA Regulatory Network Mediates Activation of mTOR and VEGF Signaling in African American Prostate Cancer

**DOI:** 10.3390/ijms23062926

**Published:** 2022-03-08

**Authors:** Himali Gujrati, Siyoung Ha, Azah Mohamed, Bi-Dar Wang

**Affiliations:** 1 Department of Pharmaceutical Sciences, School of Pharmacy, University of Maryland Eastern Shore, Princess Anne, MD 21853, USA; hgujarati@umes.edu (H.G.); gktldud888@gmail.com (S.H.); 2 Toxicology Program, University of Maryland Eastern Shore, Princess Anne, MD 21853, USA; amabdallamohamed@umes.edu; 3 Hormone Related Cancers Program, University of Maryland Greenebaum Comprehensive Cancer Center, Baltimore, MD 21201, USA

**Keywords:** prostate cancer disparities, microRNA, reciprocal miRNA-mRNA pairing, precision biomarker, therapeutic strategy

## Abstract

African American (AA) men exhibit 1.6-fold higher prostate cancer (PCa) incidence and 2.4-fold higher mortality rates compared to European American (EA) men. In addition to socioeconomic factors, emerging evidence suggests that intrinsic biological differences may explain part of PCa disparities. In this study, we applied microRNA (miRNA)-driven bioinformatics to evaluate whether differential miRNA-mRNA regulatory networks play a role in promoting the AA PCa disparities. 10 differentially expressed miRNAs were imported to mirPath V.3 algorithm, leading to identification of 58 signaling pathways differentially regulated in AA PCa versus EA PCa. Among these pathways, we particularly focused on mTOR and VEGF signaling, where we identified 5 reciprocal miRNA-mRNA pairings: miR-34a-5p/*HIF1A,* miR-34a-5p/*PIK3CB*, miR-34a-5p/*IGFBP2*, miR-99b-5p/*MTOR* and miR-96-5p/*MAPKAPK2* in AA PCa versus EA PCa. RT-qPCR validation confirmed that miR-34a-5p, miR-99b-5p and *MAPKAPK2* were downregulated, while miR-96-5p, *IGFBP2*, *HIF1A*, *PIK3CB* and *MTOR* were upregulated in AA PCa versus EA PCa cells. Transfection of miRNA mimics/antagomir followed by RT-qPCR and Western blot analysis further verified that *IGFBP2*, *HIF1A* and *PIK3CB* are negatively regulated by miR-34a-5p, whereas *MTOR* and *MAPKAPK2* are negatively regulated by miR-99b-5p and miR-96-5p, respectively, at mRNA and protein levels. Targeting reciprocal pairings by miR-34a-5p mimic, miR-99b-5p mimic or miR-96-5p antagomir downregulates HIF1α, PI3Kβ, mTOR, IGFBP2 but upregulates MAPKAPK2, subsequently reducing cell proliferation and sensitizing docetaxel-induced cytotoxicity in PCa cells. These results suggest that miRNA-mRNA regulatory network plays a critical role in AA PCa disparities, and targeting these core miRNA-mRNA pairings may reduce PCa aggressiveness and overcome the chemoresistance in AA patients.

## 1. Introduction

Human microRNAs (miRNAs) are ~22 nucleotide (nt) non-coding RNAs, involved in the regulation of post-transcriptional gene expression profile. It is estimated that more than 60% of human protein coding genes are regulated by the miRNAs [1,2]. MiRNAs play critical roles in biological processes such as cell proliferation, cell growth, intracellular signaling, cell differentiation, cell apoptosis, cellular metabolism and carcinogenesis [3,4]. They usually bind to the 3′-untranslated region (3′-UTR) of the target mRNAs, destabilizing the mRNA and control protein production through translational silencing [5]. The genes encoding miRNAs locate in exonic or intronic regions and are transcribed by RNA polymerase II, resulting in pri-miRNAs. The pri-miRNAs are further processed by Drosha complex to form the ~70 nt stem-loop pre-miRNAs [6]. Exportin-5 and Ran-GTP transports pre-miRNAs from the nucleus to cytoplasm, where Dicer further processes pre-miRNAs into 20–25 nucleotide long mature miRNA-miRNA duplexes. These mature miRNAs are then loaded onto Argonaute 2 protein (AGO2) and RNA-induced silencing complex (RISC) to achieve site-specific cleavage/degradation or translational inhibition of the target mRNAs [7,8].

Prostate cancer (PCa) is now the most frequently diagnosed cancer and the second leading cause of cancer deaths among American men [9]. Notably, African Americans (AAs) are 1.6 times more likely to develop PCa, and 2.4 times more likely to die from this disease compared to their European American (EA) counterparts [9]. Multiple socioeconomic factors have been postulated to explain the observed PCa disparities [10,11]. However, higher mortality and recurrence is still observed in AAs after adjustment for socioeconomic status [12,13], indicating that intrinsic biological differences account for at least part of the PCa disparities [10,13,14].

MiRNAs play important roles in either promoting or preventing cancer development and/or progression. MiRNAs promoting tumorigenesis are referred as oncogenic miRNAs or ‘oncomirs’, while miRNAs function as tumor suppressor are termed tumor suppressive miRNAs [15,16]. Accumulating evidence indicates that miRNAs and their regulatory and biogenesis mechanisms are involved in the development of prostate cancer. Previous studies have shown that let-7a, let-7c, miR-15a, miR-20, miR-24, miR-29, miR-125b, miR-128a, miR-143, miR-145, miR-181a, miR-181b, and miR-222 are downregulated in both PCa cell lines and/or tissues [17,18,19,20,21], whereas let-7d*, miR-17-5p, miR-21, miR-141, miR-148a, miR-182, miR-200b, miR-200c, and miR-375 are upregulated in PCa cells and/or tissues [18,20,21]. Recent studies further shed light on deciphering the miRNA-mediated mechanisms underlying PCa disparities. For instance, overexpression of miR-130b has been linked to PCa aggressiveness and poor clinical outcome in AA [22]. Previous study has also shown that miR-182 is upregulated in AA PCa vs. EA PCa, and the high-level miR-182 is correlated with poorer survival rate in AA PCa [23]. In addition, downregulation of miR-34b and miR-146a have been observed in AA PCa vs. EA PCa [24,25]. AR and ETV1 were shown to be directly targeted by miR-34b, and the downregulation of miR-34b enhances AR expression and promotes cell proliferation in AA PCa [25]. Moreover, we previously employed a systems biology approach to evaluate the functional impacts of population-associated miRNAs and mRNAs in a three-way comparison in PCa (AA PCa vs. EA PCa, AA PCa vs. AA normal, EA PCa vs. EA normal). ErbB signaling has been identified as a critical cascade significantly upregulated by miRNA-mRNA regulatory network in AA PCa [26]. In this study, we revisited our genomic data and particularly focused on the 10 differently expressed miRNAs identified between AA and EA PCa, to further assess their functional impacts in AA PCa disparities. Specifically, we performed an integrative genomic approach, combining a miRNA-driven pathway analysis algorithm with miRNA target prediction and unique mRNA mapping, to identify cancer signaling pathways significantly influenced by the miRNA-mRNA regulatory network in AA PCa. Through this analysis, we have identified 58 significant pathways regulated by miRNAs and their target mRNAs in AA PCa. Notably, ErbB-PI3K-AKT-mTOR-HIF-VEGF axis was identified as central signaling cascade highly regulated by AA-depleted/enriched miRNAs and mRNAs in AA PCa. A novel panel of reciprocal miRNA-mRNA pairings were defined as core miRNA-mRNA regulatory components within mTOR and VEGF signaling, potentially serving as potential precision biomarkers and novel therapeutic targets for AA PCa.

## 2. Results

### 2.1. MirPath v.3 Analysis Reveals 68 Signaling Pathways Differentially Regulated by miRNA-mRNA Networks in AA PCa and EA PCa

In previous genomic studies, total RNA samples isolated from 20 AA and 15 EA PCa specimens were subjected to miRNA profiling [26] and mRNA profiling [26,27] analysis. 10 miRNAs were found differentially expressed between AA and EA PCa, including 8 AA-depleted miRNAs (downregulated in AA PCa vs. EA PCa) and 2 AA-enriched miRNAs (upregulated in AA PCa vs. EA PCa) (Supplemental Table S1) [26]. By importing these 10 AA-depleted/enriched miRNAs into the mirPath v.3 algorithm [28], 58 KEGG pathways were identified as significant pathways (adjusted *p*-value < 0.05) differentially regulated between AA PCa and EA PCa (Table 1). Several canonical oncogenic signaling pathways (such as ErbB, mTOR, HIF-1, VEGF and focal adhesion pathways) were significantly influenced by the AA-depleted/enriched miRNAs (Table 1). In addition, critical signaling pathways related to cancer, cell cycle, inflammation and cell death regulations (such as p53, cell cycle, insulin, NF-kappa B and TNF signaling) were also differentially regulated in AA PCa vs. EA PCa (Table 1).

### 2.2. The mTOR and VEGF Signaling Pathways Are Upregulated in AA PCa Compared to EA PCa

**Figure 1:**
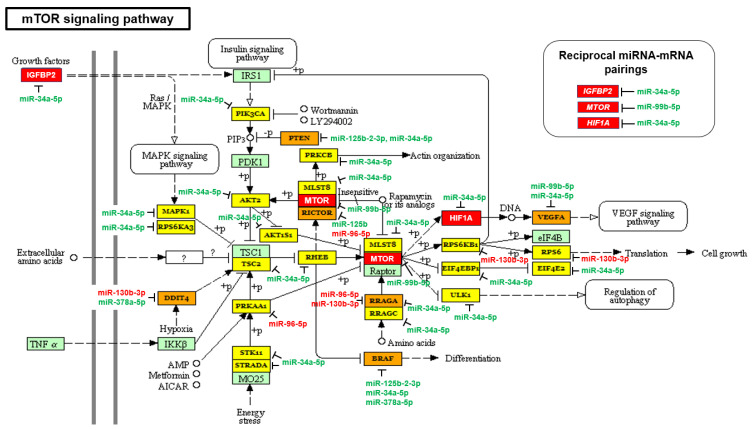
The mTOR signaling pathway is upregulated in AA prostate cancer (PCa) specimens. MirPath v.3 was used to evaluate the impact of the 10 differentially expressed miRNAs (in AA PCa vs. EA PCa) in regulating the biological signaling pathways in AA PCa compared to EA PCa. Downregulated (green) and upregulated (red) miRNAs and mRNAs, in AA PCa vs. EA PCa, were indicated in mTOR signaling pathway. Three robust reciprocal miRNA-mRNA pairings, *IGFBP2*/miR-34a-5p (up/down), *MTOR*/miR-99b-5p (up/down) and *HIF1A*/miR-34a-5p (up/down), were highlighted. Unpaired AA-depleted miRNAs (miR-125b-2-3p, miR-378a-5p and miR-34a-5p) and AA-enriched miRNAs (miR-130b-3p and miR-96-5p) were indicated adjacent to their predicted target genes. The genes highlighted with yellow are genes targeted by one AA-depleted/enriched miRNA. The genes highlighted with orange are genes targeted by at least two AA-depleted/enriched miRNAs. The genes highlighted with light green are genes not targeted by any AA-depleted/enriched miRNA.

**Figure 2:**
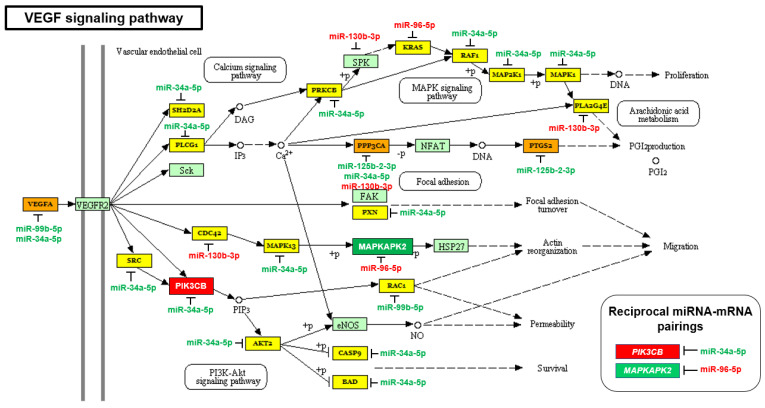
VEGF signaling pathway is upregulated in AA PCa specimens. Using mirPath v.3, VEGF signaling was identified as significant signaling pathway differentially regulated by AA depleted/enriched miRNAs (adjusted *p*-value = 0.0155). Downregulated (green) and upregulated (red) miRNAs and mRNAs, in AA PCa vs. EA PCa, were mapped in VEGF signaling pathway. Two reciprocal miRNA-mRNA pairings, *PIK3CB*/miR-34a-5p (up/down) and *MAPKAPK2*/miR-96-5p (down/up), were highlighted. Other unpaired downregulated and upregulated miRNAs (miR-125b-2-3p, miR-99b-5p and upregulated miR-130b-3p) were indicated next to their predicted target genes. The genes highlighted with yellow are genes targeted by one AA-depleted/enriched miRNA. The genes highlighted with orange are genes targeted by at least two AA-depleted/enriched miRNAs. The genes highlighted with light green are genes not targeted by any AA-depleted/enriched miRNA.

Previously, we identified ErbB signaling pathway, by employing global test using mRNA profiling data and coupling with miRNA mapping, as a significant pathway that is highly activated by miRNA-mRNA interaction in AA PCa [26]. In this study, we first performed the miRNA-driven pathway analysis using mirPath v3 [28] then followed by mapping of differentially expressed mRNAs [26] in the identified pathways. Consistent with the previous finding [26], ErbB signaling ranks as a top canonical pathway significantly regulated by AA depleted/enriched miRNAs and mRNAs (adjusted *p*-value = 1.35 × 10^−5^, Table 1). Notably, downstream of ErbB (or EGFR) signaling, the mTOR, HIF1A and VEGF signaling were also identified as significant signaling pathways influenced by AA depleted/enriched miRNAs and mRNAs (Table 1). These results suggest that ErbB (EGFR)-mTOR-HIF1A-VEGF axis as a critical signaling cascade mediated by miRNA-mRNA regulatory network in AA PCa. To understand the miRNA-mRNA regulation in this signaling cascade, we conducted a detailed miRNA/mRNA mapping in mTOR (HIF1A is part of the KEGG mTOR signaling) and VEGF signaling in AA PCa vs. EA PCa. In KEGG mTOR signaling (Figure 1), three oncogenes (*IGFBP2*, *MTOR* and *HIF1A*) were upregulated in AA PCa vs. EA PCa. The majority of KEGG mTOR signaling components (25 out of 38 genes) were predicted to be targeted by AA-depleted miRNAs (miR-34a-5p, miR-99b-5p, miR-125b-2-3p and miR-378a-5p, which were downregulated in AA PCa vs. EA PCa), and only five genes were predicted to be targeted by AA-enriched miRNAs (miR-96-5p and miR-130b-3p, which were upregulated in AA PCa vs. EA PCa). From the observed miRNA/mRNA mapping results, the mTOR signaling is theoretically upregulated in AA PCa compared to EA PCa. Similarly, 60% of VEGF signaling components (17 out of 28 genes) were predicted to be targeted by AA-depleted miRNAs (miR-34a-5p and miR-125b-2-3p), and six genes targeted by AA-enriched miRNAs (miR-96-5p and miR-130b-3p) (Figure 2). These results implicate that VEGF signaling is preferably upregulated in AA PCa when compared to EA PCa. As noted in our previous study, reciprocal miRNA-mRNA pairings defined from miRNA and mRNA profiling data (down/up or up/down in miRNA/mRNA expression in AA PCa vs. EA PCa) represent the most robust miRNA-mRNA interaction that can be experimentally validated [26]. In the mTOR and VEGF signaling pathways, we have identified five miRNA-mRNA reciprocal pairings in AA PCa. These miRNA-mRNA pairings included: miR-34a-5p/*IGFBP2* (down/up), miR-34a-5p/*HIF1A* (down/up), miR-34a-5p/*PIK3CB* (down/up), miR-99b-5p/*MTOR* (down/up), and miR-96-5p/*MAPKAPK2* (up/down) (Figure 1 and Figure 2).

### 2.3. RT-qPCR Validations and Western Blot Analysis for the Candidate Reciprocal miRNA-mRNA Pairings in EA and AA PCa Tissues and Cell Models

**Figure 3:**
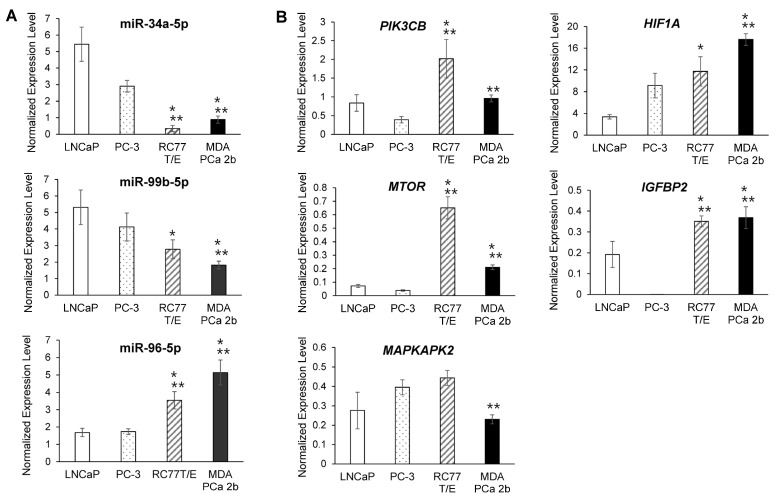
RT-qPCR validation of differentially expressed miRNAs and mRNAs in AA and EA PCa cell line models. (**A**) RT-qPCR assays for examining expression level of miR-34a-5p, miR-99b-5p and miR-96-5p in cell lines derived from EA PCa (LNCaP and PC-3) and AA PCa (RC77 T/E and MDA PCa 2b). (**B**) RT-qPCR assays for examining expression level of *PIK3CB*, *MTOR*, *HIF1A*, *IGFBP2* and *MAPKAPK2* in AA and EA PCa cell line models. Data were presented as mean ± SEM of *n* = 4-6 independent experiments, with technical triplicates for each independent experiment. The significance (* *p* < 0.05 in AA PCa cell line vs. LNCaP, and ** *p* < 0.05 in AA PCa cell line vs. PC-3) was determined based on ANOVA with Tukey post hoc test.

**Figure 4:**
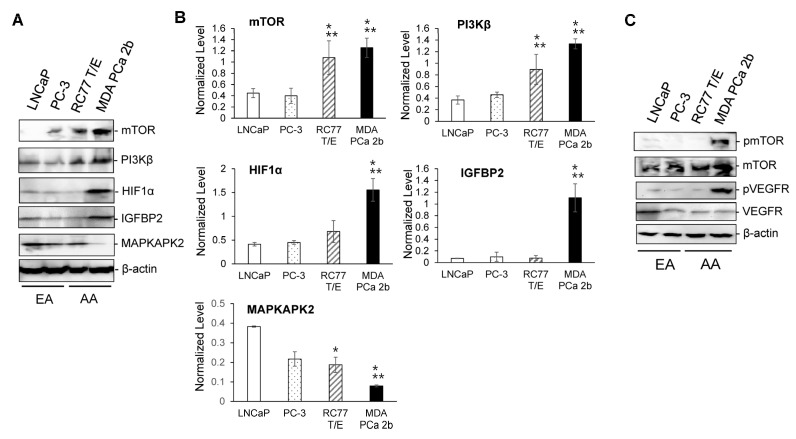
Western blot analysis of mTOR, PI3Kβ, HIF1α, IGFBP2 and MAPKAPK2 in AA and EA PCa cell line models. (**A**) Representative Western blot images of mTOR, PI3Kβ, HIF1α, IGFBP2, MAPKAPK2 and β-actin in EA PCa (LNCaP, PC-3) and AA PCa (RC77 T/E and MDA PCa 2b) cell lines. (**B**) Normalized protein levels of mTOR, PI3Kβ, HIF1α, IGFBP2 and MAPKAPK2 in EA PCa and AA PCa cell line models. The normalized protein level was measured by dividing intensity of the tested protein (mTOR, PI3Kβ, HIF1α, IGFBP2 or MAPKAPK2) with intensity of endogenous protein β-actin. Data were presented as mean ± SD of *n* = 3–4 independent immunoblot experiments, and the significance (* *p* < 0.05 in AA PCa cell line vs. LNCaP, and ** *p* < 0.05 in AA PCa vs. PC-3) was determined based on ANOVA with Tukey post hoc test. (**C**) Phosphorylation states of mTOR and VEGF in EA PCa (LNCaP and PC-3) and AA PCa (RC77 T/E and MDA PCa 2b) cell models. The pmTOR/mTOR and pVEGFR/VEGFR ratios were significantly higher in MDA PCa 2b (a metastatic AA PCa line) than in other three cell lines.

To verify the expression level of the candidate miRNA-mRNA pairings, RT-qPCR assays were performed to examine the miRNA and mRNA expression levels in EA PCa and AA PCa patient specimens and cell line models. RNA samples from 11 EA PCa and 10 AA PCa needle biopsy specimens were subjected to RT-qPCR validation of miR-34a-5p, miR-99b-5p, and miR-96-5p expression levels. The RT-qPCR validation confirmed that miR-34a-5p and miR-99b-5p were downregulated, while miR-96-5p was upregulated in AA PCa vs. EA PCa (Supplemental Figure S1), which is consistent with the miRNA array data [26]. To further elucidate the functional impacts of these AA-depleted/enriched miRNAs in PCa disparities, four PCa cell lines derived from EA and AA patients were used as *in-vitro* cell line models. LNCaP and PC-3, lymph node and bone metastasis derived from EA patients respectively, were used as metastatic EA PCa cell models. RC77 T/E, a primary PCa derived from AA patient, represents a primary AA PCa. While MDA PCa 2b, a bone metastasis derived from AA PCa patient, was used as a metastatic AA PCa cell model. To verify whether the proposed PCa cell lines can serve as in-vitro EA and AA cell models, RT-qPCR assays of AA-depleted/enriched miRNAs and mRNAs were performed. The RT-qPCR results from these EA and AA PCa cells have again confirmed the microarray data [26] and the RT-qPCR results (Supplemental Figure S1) from EA and AA PCa patient samples. Consistent with RT-qPCR results from patient samples, miR-34a-5p and miR-99b-5p were downregulated in AA PCa vs. EA PCa cell lines (Figure 3A), while their predicted targets *PIK3CB*, *MTOR*, *HIF1A* and *IGFBP2* were upregulated in AA PCa vs. EA PCa cells (Figure 3B). In contrast, RT-qPCR results confirmed that miR-96-5p was upregulated, while the its predicted target *MAPKAPK2* was downregulated in AA PCa vs. EA PCa cells (Figure 3A,B). Western blot analysis was performed to assess the protein levels of mTOR, PI3Kβ, HIF1α, IGFBP2 and MAPKAPK2. Immunoblot analysis again confirmed that mTOR, PI3Kβ, HIF1α, and IGFBP2 were significantly upregulated, while MAPKAPK2 was downregulated in AA PCa (especially in MDA PCa 2b) in comparison with EA PCa cell lines (Figure 4A,B). Furthermore, immunoblot analysis was performed to assess the activation status of mTOR and VEGF signaling in EA and AA PCa cells. The Western blot assays have revealed increased phosphorylation states of mTOR and VEGF in the metastatic AA PCa cell line MDA PCa 2b, but not in metastatic EA PCa cell line (LNCaP and PC-3) and primary AA PCa cell line RC77 T/E (Figure 4C). These results, again, confirmed that mTOR and VEGF signaling pathways are preferentially upregulated in metastatic AA PCa vs. metastatic EA PCa.

### 2.4. Assessment of Regulatory Relationship in Reciprocal miRNA-mRNA Pairings by Modulating AA-Depleted or AA-Enriched miRNA Expression Level

**Figure 5:**
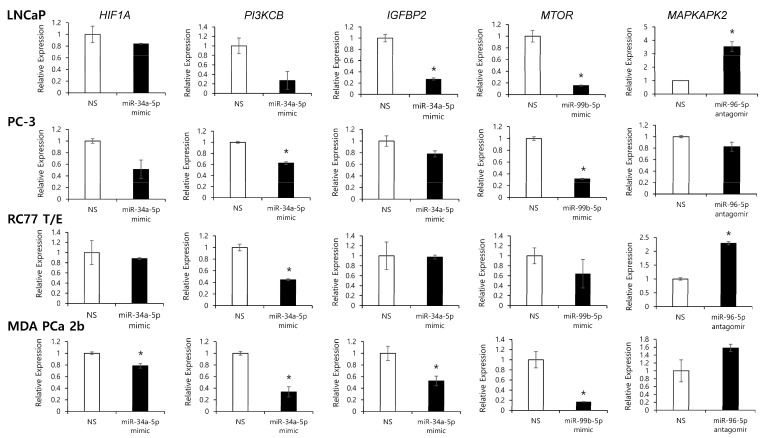
Modulation of miRNA expression affects the transcriptional regulation of its target genes in EA and AA PCa cell lines. RT-qPCR analysis of *MTOR*, *PIK3CB*, *HIF1A*, *IGFBP2* and *MAPKAPK2* expression in EA and AA PCa cell line models. Relative expression levels of *MTOR*, *PIK3CB*, *HIF1A*, *IGFBP2* and *MAPKAPK2* were shown in EA PCa (LNCaP and PC-3) and AA PCa (RC77 T/E and MDA PCa 2b) cell lines transfected with nonsense scrambled miRNA (NS), miRNA mimic or antagomir (miR-34a-5p mimic, miR-99b-5p mimic, or miR-96-5p antagomir). Data were presented as mean ± SEM of *n* = 3–4 independent RT-qPCR experiments, with technical triplicates for each independent experiment. The significance (* *p* < 0.05 in miRNA mimic or antagomir vs. NS) was determined based on student’s *t*-test.

**Figure 6:**
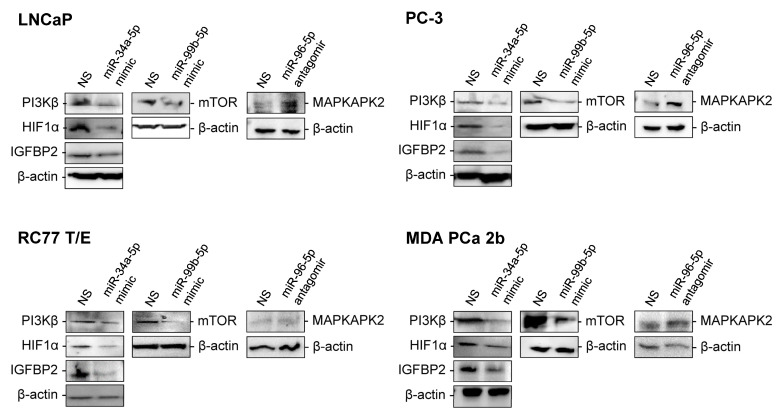
Western blot analysis of mTOR, PI3Kβ, HIF1α, IGFBP2 and MAPKAPK2 in EA and AA PCa cell line models transfected with miRNA mimics or antagomir. Protein levels of mTOR, PI3Kβ, HIF1α, IGFBP2, MAPKAPK2 and β-actin were shown in EA PCa (LNCaP and PC-3) and AA PCa (RC77 T/E and MDA PCa 2b) cell lines transfected with nonsense scrambled miRNA (NS), miR-34a-5p mimic, miR-99b-5p mimic, or miR-96-5p antagomir. Representative Western blot images were selected from 3–4 independent immunoblot assays with consistent results.

To further verify regulatory relationship in these reciprocal miRNA-mRNA pairings, EA and AA PCa cell lines were transfected with miR-34a-5p mimic, miR-99b-5p mimic or miR-96-5p antagomir then followed by RT-qPCR and Western blot analysis to examine the expression of the predicted miRNA target genes (miR-34a-5p targets genes *PIK3CB*, *HIF1A*, and *IGFBP2*; miR-99b-5p target gene *MTOR*; and miR-96-5p target gene *MAPKAPK2*) at mRNA and protein levels. Note that similar transfection efficiencies of miRNA mimics or antagomir in four PCa cell lines were confirmed by RT-qPCR assays (Supplemental Figure S2). As anticipated, transfection of miR-34a-5p mimic and miR-99b-5p mimic resulted in decreased *PIK3CB*, *HIF1A*, *IGFBP2* and *MTOR* expression in AA and EA PCa cells when compared to PCa cells transfected with nonsense/scrambled (NS) miRNA control (Figure 5). Whereas, transfection of miR-96-5p antagomir increased the expression of *MAPKAPK2* transcript in LNCaP, RC77 T/E and MDA PCa 2b cells when compared to the NS-transfected cells (Figure 5). Since miRNA targeting causes either mRNA degradation or translational repression, we further investigated the protein levels of mTOR, PI3Kβ, HIF1α, IGFBP2 and MAPKAPK2 in PCa cells transfected with NS, miR-34a-5p mimic, miR-99b-5p mimic, or miR-96-5p antagomir. As shown in Figure 6, PI3Kβ, HIF1α, and IGFBP2 were downregulated in miR-34a-5p mimic transfected vs. NS transfected PCa cells. Similarly, mTOR level was downregulated in PCa cells with miR-99b-5p mimic transfection vs. NS transfection. In contrast, MAPKAPK2 was upregulated in miR-96-5p antagomir transfected vs. NS transfected cells (Figure 6). Taken together, the RT-qPCR and immunoblot assays confirmed that *PIK3CB*, *HIF1A* and *IGFBP2* expressions are negatively regulated by miR-34a-5p, while *MTOR* and *MAPKAPK2* are negatively regulated by miR-99b-5p and miR-96-5p, respectively.

### 2.5. Modulating Expression Profile of Candidate Reciprocal miRNA-mRNA Pairings Inhibits Cell Proliferation and Sensitizes Docetaxel-Induced Cytotoxicity in PCa Cells

**Figure 7:**
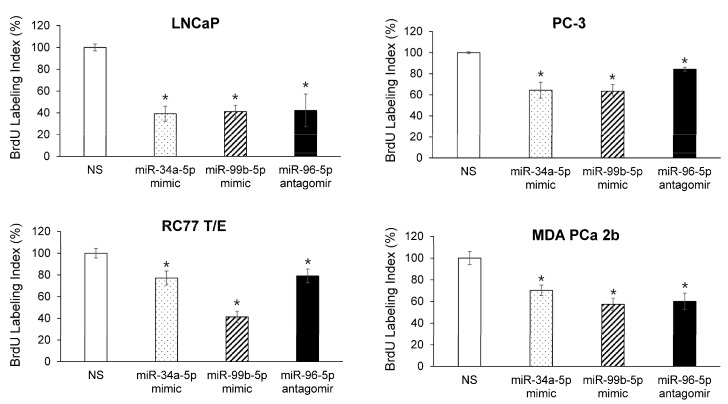
Overexpressing AA-depleted miRNAs or suppressing AA-enrich miRNA causes inhibition of cell proliferation in EA and AA PCa cell lines. BrdU-labeling assays were performed after PCa cell lines were transfected with NS, miR-34a-5p mimic, miR-99b-5p mimic or miR-96-5p antagomir for 48 h. Data were plotted as mean ± SEM of *n* = 3–4 independent assays, with 3–4 technical replicates for each independent assay. The significance (* *p* < 0.05 in miRNA mimic/antagomir vs. NS) was determined based on ANOVA with Dunnett post hoc test.

To further test the causal link among the reciprocal miRNA-mRNA pairings identified from mTOR and VEGF signaling in AA PCa (Figure 1 and Figure 2), miR-34a-5p mimic, miR-99b-5p and miR-96-5p were transfected into EA and AA PCa cells followed by in-vitro functional assays to examine their effects on cell proliferation and apoptosis initiation. Transfection of miR-34a-5p or miR-99b-5p resulted in the reduction of cell proliferation in EA PCa (LNCaP and PC-3) and AA PCa (RC77 T/E and MDA PCa 2b) cell lines (Figure 7). Conversely, transfection of miR-96-5p antagomir increased the MAPKAPK2 expression (Figure 6) and subsequently suppressed the cell proliferation in EA and AA PCa cells (Figure 7).

**Figure 8:**
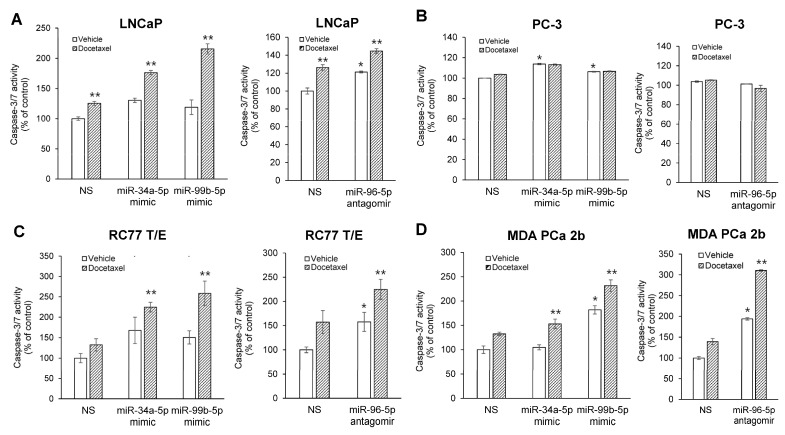
Overexpression of miR-34a-5p mimic, miR-99b-5p mimic or miR-96-5p antagomir enhances docetaxel-induced cytotoxicity in EA PCa (**A**,**B**) and AA PCa (**C**,**D**) cell lines. Apoptosis activity was assessed by measuring caspase-3/7 activity using the Apo-ONE Kit, and the data were normalized to caspase-3/7 level of vehicle-treated NS control. Data were plotted as mean ± SEM for *n* of 3–4 independent experiments, with technical triplicates for each independent experiment. The significance (* *p* < 0.05 in miRNA mimic or antagomir transfection plus vehicle treatment vs. NS plus vehicle treatment, and ** *p <* 0.05 in miRNA mimic or antagomir transfection plus docetaxel treatment to miRNA mimic or antagomir transfection with vehicle treatment) was determined using ANOVA with Tukey post hoc test.

Next, we tested whether these miRNA mimics and antagomir functionally promote cell apoptosis in the absence and presence of docetaxel, a chemotherapeutic agent commonly used in PCa treatment [29]. Specifically, the EA PCa (LNCaP and PC-3) and AA PCa (RC77 T/E and MDA PCa 2b) cell lines were transfected with NS or miRNA mimic/antagomir in the presence of vehicle or docetaxel treatment, then followed by caspase 3/7 activity assays to measure the apoptosis capacity. In the absence of docetaxel (vehicle control), transfection of PCa cell lines with miR-34a-5p mimic, miR-99b-5p mimic, or miR-96-5p antagomir caused an overall generalized or significant increase (except the miR-96-5p antagomir transfected PC-3) in cell apoptosis compared with NS transfected cells (Figure 8). Docetaxel treatment alone (without miRNA mimic/antagomir transfection) significantly induced apoptosis in EA cell line LNCaP (Figure 8A), but not in androgen-independent PC-3 (Figure 8B) and AA PCa lines (RC77 T/E and MDA PCa 2b, Figure 8C,D). As anticipated, the combination of docetaxel with miR-34a-5p mimic, miR-99b-5p mimic, or miR-96-5p antagomir further enhanced the docetaxel-induced cytotoxicity in LNCaP cells (Figure 8A). Interestingly, in the absence of miRNA mimic or antagomir transfection, AA PCa cell lines demonstrated chemoresistance to docetaxel treatment (Figure 8C,D, docetaxel vs. vehicle in NS transfected cells). In contrast, transfection of miR-34a-5p mimic, miR-99b-5p mimic, or miR-96-5p antagomir in the presence of docetaxel significantly induced apoptosis in the AA cell lines RC77 T/E and MDA PCa 2b (Figure 8C,D). These results strongly suggest that modulating AA-depleted/enriched miRNAs (i.e., overexpressing miR-34a-5p, miR-99b-5p, or inhibiting miR-96-5p) effectively sensitizes the docetaxel-induced cytotoxicity, especially in AA PCa cells.

## 3. Discussion

In this study, we utilized an integrated genomic approach in combination with a miRNA-driven computational algorithm to identify the KEGG pathways significantly influenced by the AA-depleted/enriched miRNAs and mRNAs. ErbB-PI3K-AKT-mTOR-HIF1A-VEGF signaling was identified as a critical cascade highly mediated by miRNA-mRNA regulatory network in AA PCa. A novel panel of reciprocal miRNA-mRNA pairings (miR-34a-5p/*HIF1A*, miR-34a-5p/*IGFBP2*, miR-34a-5p/*PIK3CB*, miR-99b-5p/*MTOR*, and miR-96-5p/*MAPKAPK2*) were identified as core regulators contributing to the upregulation of mTOR and VEGF signaling in AA PCa. By applying molecular and biochemical approaches, we have confirmed that *HIF1A*, *IGFBP2* and *PIK3CB* is negatively regulated by miR-34a-5p, while *MTOR* and *MAPKAPK2* are negatively regulated by miR-99b-5p and miR-96-5p, respectively. Furthermore, functional assays have validated that modulating expression profile of miR-34a-5p/*HIF1A*, miR-34a-5p/*IGFBP2*, miR-34a-5p/*PIK3CB*, miR-99b-5p/*MTOR*, or miR-96-5p/*MAPKAPK2* using miRNA mimics/antagomir subsequently inhibits cell proliferation and promotes docetaxel-induced cytotoxicity in PCa cells. Especially, AA PCa cells (more resistant to docetaxel treatment than EA PCa cells) demonstrated higher sensitivity to the treatment when combining miRNA mimics/antagomir (miR-34a-5p mimic, miR-99b-5p mimic, or miR-96-5p) with docetaxel. These results suggest that targeting these reciprocal miRNA-mRNA pairings may overcome chemoresistance and greatly sensitizes docetaxel-induced cytotoxicity, potentially serving as novel synergistic therapeutics for treating AA PCa.

Previous studies have demonstrated the critical functional roles of miR-34a-5p and miR-99b-5p as tumor suppressive miRNAs, and miR-96-5p as an oncogenic miRNA in various types of cancers. Downregulation of miR-34a-5p has been implicated in pancreatic cancer [30], hepatocellular carcinoma (HCC) [31], and PCa [32]. Furthermore, overexpression of miR-34a-5p resulted in significant decrease in cell proliferation and migration, and significant enhancement of cell apoptosis in HCC [31] and PCa cells [33]. Belonging to miR-125a-let-7e cluster family, miR-99b-5p has been reported to play a role in cell proliferation, migration, and differentiation in tumor cells [34]. A study by Shi et al. showed a significant reduction of miR-99b-5p expression in osteosarcoma (OS) tissue/cell lines compared to normal tissues/cells, suggesting it could potentially serve as a biomarker for OS patients [35]. MiR-96-5p belongs to the cluster miR-183-96-182 family and has been found upregulated in various cancers including breast cancer, thyroid cancer, bladder cancer, adrenocortical and adrenal medullary tumors, head, neck squamous cell carcinoma, and cervical cancer [36,37]. Furthermore, high-level expression of miR-96-5p is correlated with a poor overall survival rate of PCa patients, and has been implicated in promoting PCa growth, proliferation and tumor progression [38].

Hypoxia-inducible factors (HIFs) act as a driving force for cancer cells to adapt hypoxic condition, which contributes to cancer progression and treatment resistance [39]. Previous study further revealed androgen receptor (AR)-hypoxia-HIF1α axis as an independent pathway to promote PCa development [40]. It has been reported that *HIF1A* is a direct mRNA target of miR-34a-5p, and overexpression of miR-34a-5p downregulates HIF1α and other epithelial-to-mesenchymal transition (EMT) markers, consequently inhibiting VEGFR signaling in breast cancer [41]. Another study also indicated that miR-34a-5p directly targets *HIF1A* and inhibits its transcription, preventing PPP1R11/STAT3-induced EMT and metastasis in colorectal cancer (CRC) [42].To our knowledge, our study is the first report to confirm that transcription and translation of *HIF1A* is also negatively regulated by miR-34a-5p in PCa (Figure 5 and Figure 6). Taken together, p53-miR-34a-5p-HIF1α signaling axis may play a critical role in pathogenesis of PCa and other cancers. Therefore, targeting miR-34a-5p-*HIF1A* pairing (i.e., using miR-34a-5p mimic or HIF1α inhibitor) may serve as an effective molecular strategy to reduce PCa aggressiveness, especially in AA patients.

Insulin-like growth factor binding protein 2 (IGFBP2) is an oncogenic protein involved in the development and progression of various cancers, such as glioma, breast, lung and prostate cancers [43]. In addition, IGFBP2 has been shown to overexpress in PCa and prostatic intraepithelial neoplasia (PIN) tissues [44]. PCa cells produce significantly high amount of IGFBP2, and the serum level of IGFBP2 is positively correlated with the tumor grades and stages, suggesting the potential role of IGFBP2 as a biomarker in PCa risk, resistance and relapse [45]. Furthermore, *IGFBP2* has been uncovered as a direct target of miR-34a-5p, and miR-34a-5p expression inhibits the expression of *IGFBP2* at transcriptional and translational levels in myoblasts [46]. Our results also confirmed, as the first time, that *IGFBP2* is negatively regulated by miR-34a-5p at transcriptional and translation levels in PCa cells (Figure 5 and Figure 6). These results suggest miR-34a-5p/*IGFBP2* pairing as a potential precision biomarker and novel drug target in PCa.

Excessive activity in the PI3K/AKT/mTOR signaling pathway, one of the most common hallmarks in human cancers, is an important therapeutic target for cancer treatment. Aberration of p110β (PI3Kβ) and its overexpression has been implicated in carcinogenesis process in different cancers [47]. Previous study revealed that miR-34a-5p directly targets/inhibits *PIK3CB* and participates in the regulation of TCR-mediated NFκB signaling in CD4^+^ and CD8^+^ T cells [48]. Similarly, our data confirmed, as the first time, that *PIK3CB* is negatively regulated by miR-34a-5p at mRNA and protein levels in PCa (Figure 5 and Figure 6), indicating miR-34a-5p/*PIK3CB* reciprocal pairing as a potential biomarker as well as novel therapeutic target in AA PCa.

mTOR is a downstream target of PI3K/AKT survival pathways and functions as a regulator involved in cell growth, proliferation, and survival [49]. Deregulation of mTOR contributes to cancer progression and drug resistance [50]. MiR-99a/b has been implicated as a tumor suppressor frequently downregulated in human cancers, and plays an important role in regulating mTOR signaling pathway [51]. Several studies have shown that downregulation of miR-99b-5p is correlated with the elevated levels of mTOR in PCa and endometrial carcinoma [52]. Previous study has further revealed that miR-99b-5 expression suppresses mRNA and protein levels of mTOR, PI3K, AKT and p70S6, thereby inhibiting PI3K/AKT/mTOR signaling in human cervical cancer [53]. Upregulation of miR-99b-5p results in a longer survival, while silencing of miR-99b-5p causes upregulation of mTOR and promotes cell migration in CRC [54]. MiR-99b-5p has been revealed to directly or indirectly target *MTOR*, and inhibiting miR-99b-5p expression causes upregulation of mTOR in PCa and endometrial carcinoma [55]. In addition, overexpression of miR-99a/b in HCC cells results in significant inhibition of tumor growth by suppressing the expression of *IGF1R* and *MTOR* [56]. In this study, we confirmed that miR-99b-5p functions as a tumor suppressive miRNA (Figure 7 and Figure 8), and its expression contributes to the suppression of *MTOR* expression at transcriptional and translational levels in PCa (Figure 5 and Figure 6). Given all, deregulation of miR-99-*MTOR* signaling represents a crucial miRNA-mRNA interaction in PCa development/progression. Modulation of miR-99b-5p/*MTOR* expression profile through miR-99b-5p mimic reduces the PCa aggressiveness and sensitizes docetaxel-induced cytotoxicity, especially in AA PCa.

Activation of the pathway mitogen-activated protein kinase (MAPK) is found to play a diverse role in multiple cellular mechanisms, including cell growth, migration, proliferation, differentiation, and apoptosis [57]. Mitogen-activated protein kinase 2 (MAPKAPK2, also named as MK2) is a direct downstream substrate of p38 MAPK, and it plays a critical role in regulating particular cellular functions, including apoptosis, cell cycle, DNA repair, RNA metabolism, autophagy, inflammation, post-translational regulation of gene expression, and stress response to oxidative agents [58,59]. Previous studies have shown that p38 MAPK has a dual role as a tumor suppressor kinase or a tumor promoter [59], however, the diverse functional roles of MAPKAPK2 remain elusive. Previous study has implicated MAPKAPK2 as an oncogene involved in tumorigenesis in lung, colorectal, skin, bladder, and prostate cancers [60,61]. Several reports have also demonstrated that p38 MAPK signaling functions as an antitumor pathway. p38 MAPK exerts its tumor suppressive activities by inhibiting oncogenic transformation, such as regulating cell cycle, inhibiting cell proliferation, activating cell apoptosis, inducing senescence, modulating inflammatory-dependent transformation, and promoting cell differentiation [57,59]. MAPKAPK2, a key p38 downstream substrate involved in cell cycle control, DNA repair, immune response, senescence, and autophagy [58], may also function as a cofactor in the p38-mediated tumor suppressor pathways. In this study, DIANA-TarBase algorithm predicted *MAPKAPK2* as a target of miR-96-5p, and our results have further confirmed that miR-96-5p negatively regulates *MAPKAPK2* expression at transcriptional and translational levels. To our knowledge, this is the first study to confirm that *MAPKAPK2* expression is mediated by miR-96-5p. Further luciferase reporter assay will validate whether *MAPKAPK2* is a direct or indirect miR-96-5p target. In summary, although the tumor suppressive role of *MAPKAPK2* remains elusive, our results have implicated miR-96-5p/*MAPKAPK2* as a promising therapeutic target for reducing the chemoresistance in AA PCa.

In conclusion, our study has revealed that EGFR-PI3K-ATK-mTOR-HIF1α-VEGF axis is preferentially upregulated by AA-depleted/enriched miRNAs in PCa. The novel panel of reciprocal miRNA-mRNA pairings within mTOR signaling (miR-34a-5p/*HIF1A*, miR-34a-5p/*IFGBP2*, and miR-99b-5p/*MTOR*) and VEGF signaling (miR-34a-5p/*PIK3CB*, and miR-96-5p/*MAPKAPK2*) have been suggested as critical miRNA-mRNA regulatory components in AA PCa (or more aggressive type of PCa). Further exploring a computational algorithm using combined expression profiles of these miRNA/mRNA pairings may facilitate the development of precision diagnostic/prognostic biomarkers in PCa. Finally, targeting miRNA-mRNA reciprocal pairings (i.e., using miRNA mimics/antagomirs, siRNAs, antisense oligonucleotides, or Crispr knockdown) may lead to the development of novel molecular strategies to overcome chemoresistance observed in aggressive AA PCa.

## 4. Materials and Methods

### 4.1. Pathway Analysis and Identification of miRNA-mRNA Pairings

DIANA-mirPath V.3 [28] is a web-server based miRNA pathway analysis program that provides accurate statistics by integrating prediction of miRNA targets, based on DIANA-TarBase or TargetScan algorithm, into the identification of significant KEGG pathways. 10 differentially miRNAs in AA PCa vs. EA PCa (Supplemental Table S1) [26] were imported into DIANA-mirPath V.3 program to identify the significant signaling pathways influenced by these miRNAs. Adjusted *p*-value (FDR < 0.05) was applied to identify the most significant KEGG pathways influenced by the miRNAs imported to mirPath V.3 program. The selected signaling pathways, mTOR and VEGF signaling, were further mapped with AA-depleted and -enriched mRNAs (downregulated and upregulated mRNAs in AA PCa vs. EA PCa) [26] to further define the reciprocal miRNA-mRNA pairings in the selected signaling pathways.

### 4.2. Cell Culture and TRANSFECTION

Human PCa cell lines LNCaP and PC-3, derived from lymph node metastasis and bone metastasis of Caucasian patients, and MDA PCa 2b, derived from bone metastasis of African American patient, were purchased from American Type Culture Collection (ATCC, Manassas, VA). RC77 T/E, a primary PCa cell line derived from African American patient, was kindly provided by Dr. Johng Rhim at Center for Prostate Disease Research (CPDR) in Rockville, MD. LNCaP and PC-3 were served as EA PCa cell line models, and RC77 T/E and MDA PCa 2b were served as AA PCa cell models in the study. The cells were cultured in specific cell culture media, LNCaP was cultured in RPMI with 10% fetal bovine serum (FBS), PC-3 was cultured in DMEM with 10% FBS, MDA PCa 2b were cultured in BRFF-HPC1 with 20% FBS and RC77 T/E was cultured in Keratinocyte SFM with Human Recombinant Epidermal Growth Factor (EGF 1–53) and Bovine Pituitary Extract (BPE). Cells were maintained at 37 °C in a 5% CO_2_ incubator. LNCaP, PC-3 and MDA PCa 2b cells were seeded at density 3 × 10^4^ cells/well in 6-well plates. RC77 T/E cells were seeded at density 5 × 10^4^ cells/well in 6-well plates. The cells were allowed to grow for 24 h and then were either transfected with miR-34a-5p mimic, miR-99b-5p mimic, or miR-96-5p antagomir using DharmaFECT4 transfection reagent (Dharmacon, Lafayette, CO, USA), according to the manufacturer’s protocol. After 24 h, the cells were replaced with fresh media then incubated for 24 h. miR-34a-5p mimic, miR-99b-5p mimic, miR-96-5p antagomir, and nonsense miRNA mimic and antagomir controls were purchased from Ambion (Austin, TX, USA). nonsense/scrambled miRNA mimic and antagomir were used as negative controls.

### 4.3. RT-qPCR Validation of mRNA and miRNA Expression

For the measurement of gene expression level, total RNA was isolated using miRNeasy Mini Kit (50) (Qiagen, Germantown, MD, USA) from the PCa cells used in the study. Quantification of total RNA samples were done using NanoVue Plus spectrophotometer (GE Healthcare, Wauwatosa, WI, USA). For examining mRNA expression level, 1 µg of total RNA was used as template for reverse transcription. Reverse transcription was performed using iScript Reverse Transcription Supermix kit (Bio-Rad, Hercules, CA, USA) to generate cDNA then followed by performing quantitative PCR (qPCR) assays using SsoAdvanced Universal SYBR Green supermix kit (Bio-Rad, Hercules, CA, USA). For reverse transcription of miRNAs, the miRNA samples (1 µg per sample) were used in the reverse transcription reactions using Qiagen miScriptII RT kit. Specifically, the miRNAs were poly-A tailed, and reverse transcribed using poly-T primer coupled with 3′-nucleotide code designed by Qiagen (Germantown, MD, USA). The synthesized cDNAs were mixed with universal reverse primer (Qiagen, Germantown, MD, USA), specific miRNA primer, and PowerUp SYBR Green master mix (Applied Biosystems, Waltham, MA, USA) to perform the qPCR reactions.

The qPCR reaction condition for quantification of miRNA expression levels were as follows: pre-denaturation for 5 min at 95 °C, followed by 40 standard cycles of: denaturation at 95 °C for 15 s, annealing at 55 °C for 30 s, and extension at 70 °C for 30 s. While the qPCR reaction program for quantification of mRNA level is: pre-denaturation for 5 min at 95 °C, followed by 40 standard cycles of: denaturation at 95 °C for 15 s, then annealing and extension at 60 °C for 30 s. Specific primers designed for miR-34a-5p, miR-99b-5p and miR-96-5p and their target genes *MTOR*, *PIK3CB*, *HIF1A*, *IGFBP2* and *MAPKAPK2* were used for qPCR assays (primer sequences were listed in Supplemental Table S2). RT-qPCR determination of miRNA and mRNA expression levels were performed in triplicate and normalized to levels of endogenous miR-103a-3p and housekeeping genes *EIF1AX*, respectively [26]. Normalized gene expression levels were determined using the 2^−ΔCT^ method.

### 4.4. Western Blot Analysis

For measurement of the gene expression at protein level, proteins were extracted using M-PER Mammalian Protein Extraction Reagent (Thermo Fisher Scientific, Waltham, MA, USA) with protease and phosphatase inhibitor cocktail (Thermo Fisher Scientific, Waltham, MA, USA). Protein concentrations were quantified using BCA assay kit (Thermo Fisher Scientific, Waltham, MA, USA). Equal amounts of proteins were separated by electrophoresis using Blot 4-12% Bis-Tris gels (Invitrogen, Waltham, MA, USA) and transferred onto PVDF membranes (Bio-Rad, Hercules, CA, USA). The PVDF membranes were then incubated with primary antibodies, washed three times with 1 × TBST, then incubated with secondary antibodies. Immunoblots were developed with SuperSignal ECL substrate (Thermo Fisher Scientific, Waltham, MA, USA) and visualized using ChemiDoc XRS imaging system (Bio-Rad, Hercules, CA, USA). The primary antibodies used in the study were rabbit monoclonal antibodies against mTOR, PI3Kβ, IGFBP2, HIF-1α, MAPKAPK2 and β-actin from Cell Signaling Technology (Danvers, MA, USA). The secondary antibody used in the study was an anti-rabbit IgG-HRP antibody purchased from Thermo Fisher Scientific (Waltham, MA, USA).

### 4.5. BrdU-Labeling Cell Proliferation Assay

LNCaP, PC-3, RC77 T/E and MDA PCa 2b cells were seeded at density 3 × 10^4^ cells/well in 6-well culture plates. RC77 T/E cells were seeded at density 5 × 10^4^ cells/well in 6-well culture plates. The cells were incubated for 24 h and then were either transfected with miRNA mimics or antagomir. As a control, cells were cultured with nonsense scrambled control (NS). The cells were incubated for another 24 h. After this period, cells were plated in 96-well plates, bromodeoxyuridine (BrdU) incorporation assay was performed to analyze cell proliferation and cell viability. The assay was conducted using BrdU Cell Proliferation Assay Kit (Sigma-Aldrich, St. Louis, MO, USA) as described by manufacturers. Cells were labelled with BrdU for an incubation period of 8 h in the tissue culture incubator. After incubation, the content of each well was removed and 200 µL of fixative solution was added for 30-min incubation at room temperature. This was followed by adding 100 µL of anti-BrdU antibody into each well, and the cells were further incubated for 1 h at room temperature. The plate was then washed 3 times with 1× wash buffer. 100 µL of goat anti-mouse IgG HRP conjugate was added into each well then incubated for 30 min. After washing three times with 1× wash buffer and additional washing with ddH_2_O, 100 µL of substrate solution was added and the cells were incubated in dark for 15 min or until a significant color change was observed. 100 µL of stop solution was used to stop further reaction and followed by checking absorbance at dual wavelengths of 450 nm and 540 nm using Multiskan FC microplate photometer (Thermo Scientific, Waltham, MA, USA).

### 4.6. Caspase 3/7 Activity-Based Apoptosis Assay

For the measurement of apoptosis initiation by transfection of miRNA mimic or antagomir in PCa cells, the transfected cells 5000 cells/well were seeded onto 96-well cell culture plates (Corning, Corning, NY, USA) and then incubated overnight. The miRNA mimic/antagomir transfected cells were then treated with vehicle or 11 mM of Docetaxel [26]. 24 h after the vehicle or drug treatment, the apoptosis assays were performed using Apo-ONE Caspase-3/7 Assay Kit (Promega Corporation, Madison, WI, USA) according to the protocol described by the manufacturer. 100 µL of homogeneous Caspase-3/7 reagent was added to the sample plate and incubated at room temperature for 1 h. Fluorescence was detected for measuring the apoptosis state (Caspase 3/7 activity) using Biotek Synergy HT Microplate Reader (BioTek, Winooski, VT, USA) at wavelengths of 499 nm and 521 nm for excitation and emission, respectively.

## Figures and Tables

**Table 1:** KEGG pathways differentially regulated by population-associated miRNAs between AA and EA PCa. MirPath V.3 program was used for identification of the significant signaling pathways regulated by AA-depleted and enriched miRNAs. The significance was measured based on adjusted *p*-value (FDR < 0.05) determined by statistics implemented in mirPath V.3.

KEGG Pathway	*p*-Value	#Genes	#miRNAs
MicroRNAs in cancer	3.88 × 10^−54^	100	8
Proteoglycans in cancer	8.70 × 10^−12^	99	8
Adherens junction	4.02 × 10^−10^	45	7
Viral carcinogenesis	1.29 × 10^−7^	105	8
Glioma	3.22 × 10^−7^	39	8
Transcriptional misregulation in cancer	3.22 × 10^−7^	88	8
Cell cycle	4.05 × 10^−7^	71	7
Prostate cancer	6.70 × 10^−7^	55	8
Pancreatic cancer	1.27 × 10^−6^	44	7
Bacterial invasion of epithelial cells	2.81 × 10^−6^	44	8
Colorectal cancer	4.14 × 10^−6^	40	7
Thyroid cancer	9.18 × 10^−6^	21	6
Fatty acid biosynthesis	1.35 × 10^−5^	5	3
ErbB signaling pathway	1.35 × 10^−5^	48	8
Pathways in cancer	1.89 × 10^−5^	166	8
Chronic myeloid leukemia	2.45 × 10^−5^	43	6
Central carbon metabolism in cancer	3.86 × 10^−5^	39	7
Non-small cell lung cancer	5.33 × 10^−5^	34	6
Shigellosis	8.00 × 10^−5^	37	7
Regulation of actin cytoskeleton	9.30 × 10^−5^	100	7
Renal cell carcinoma	1.14 × 10^−4^	39	6
p53 signaling pathway	1.14 × 10^−4^	42	7
Glycosphingolipid biosynthesis—lacto and neolacto series	1.16 × 10^−4^	13	5
Endometrial cancer	1.85 × 10^−4^	31	6
Bladder cancer	2.18 × 10^−4^	26	8
Hepatitis B	2.30 × 10^−4^	68	7
Other types of O-glycan biosynthesis	3.88 × 10^−4^	15	5
Alcoholism	4.33 × 10^−4^	88	8
Endocytosis	5.08 × 10^−4^	93	7
Acute myeloid leukemia	6.12 × 10^−4^	33	6
Melanoma	8.26 × 10^−4^	38	8
Neurotrophin signaling pathway	1.746 × 10^−3^	58	8
Focal adhesion	2.185 × 10^−3^	96	8
mTOR signaling pathway	2.477 × 10^−3^	33	6
HIF-1 signaling pathway	2.827 × 10^−3^	53	8
Estrogen signaling pathway	2.827 × 10^−3^	44	8
Protein processing in endoplasmic reticulum	2.962 × 10^−3^	75	7
Oocyte meiosis	2.974 × 10^−3^	55	7
N-Glycan biosynthesis	3.345 × 10^−3^	26	7
Fc gamma R-mediated phagocytosis	5.007 × 10^−3^	46	6
Hippo signaling pathway	5.356 × 10^−3^	64	7
RNA transport	5.788 × 10^−3^	76	6
Thyroid hormone signaling pathway	6.076 × 10^−^3	58	7
Progesterone-mediated oocyte maturation	1.0333 × 10^−2^	44	6
Salmonella infection	1.1305 × 10^−2^	42	6
Axon guidance	1.2697 × 10^−2^	54	6
Ubiquitin mediated proteolysis	1.2697 × 10^−2^	62	7
Oxytocin signaling pathway	1.2697 × 10^−2^	72	8
Insulin signaling pathway	1.5334 × 10^−2^	65	8
VEGF signaling pathway	1.5546 × 10^−2^	32	5
Glycosaminoglycan biosynthesis—keratan sulfate	1.9381 × 10^−2^	32	5
Prolactin signaling pathway	2.3342 × 10^−2^	35	6
Small cell lung cancer	2.7242 × 10^−2^	41	7
FoxO signaling pathway	3.0606 × 10^−2^	62	8
NF-kappa B signaling pathway	3.4130 × 10^−2^	38	7
TNF signaling pathway	3.6058 × 10^−2^	47	6
HTLV-I infection	4.0449 × 10^−2^	107	8
Choline metabolism in cancer	4.1519 × 10^−2^	46	7

## Data Availability

Not applicable.

## Supplementary Materials

The following supporting information can be downloaded at: https://www.mdpi.com/article/10.3390/ijms23062926/s1.
