# Supplementary material for: MicroRNA-mRNA Regulatory Network Mediates Activation of mTOR and VEGF Signaling in African American Prostate Cancer
Source: Int J Mol Sci. 2022 Mar 8;23(6):2926. doi: 10.3390/ijms23062926 (PMC8949405; doi:10.3390/ijms23062926)
Supplement: Supplementary file 1 [file ijms-23-02926-s001.zip › ijms-1601477-supplementary.pdf]

Table S1. 10 differentially expressed miRNAs between AA PCa and EA PCa from our previous study [1]. The AA-depleted miRNAs are defined as the miRNAs significantly downregulated in AA PCa vs. EA PCa, while AA-enriched miRNAs are defined as miRNAs significantly upregulated in AA PCa vs. EA PCa. Significance was determined based on ANOVA with FDR< 0.1 (adjust p-value) after multiple correction from miRNA microarray data. The previous miRNA IDs and current miRNA IDs (most updated IDs from miRbase, https://www.mirbase.org/) are included in this Table. The current miRNA IDs were imported to DIANA-mirPath V.3 for further pathway analysis.

**miRNA (previous ID)**	**miRNA (current ID)**	**Fold change (AA PCa vs. EA PCa)**	**Regulation (AA PCa vs. EA PCa)**	**AA-enriched or AA-depleted miRNA**	**Adjust *p*-value, <10% FDR**
hsa-miR-125b-2*	hsa-miR-125b-2-3p	-1.3902426	downregulated	AA-depleted	yes
hsa-miR-758	hsa-miR-758-3p	-1.686261	downregulated	AA-depleted	yes
hsa-miR-99b	hsa-miR-99b-5p	-1.4173684	downregulated	AA-depleted	yes
hsa-miR-133a	hsa-miR-133a-5p	-1.4290309	downregulated	AA-depleted	yes
hsa-miR-34a	hsa-miR-34a-5p	-1.4193252	downregulated	AA-depleted	yes
hsa-miR-96	hsa-miR-96-5p	1.7657517	upregulated	AA-enriched	yes
hsa-miR-130b	hsa-miR-130b-3p	1.4793279	upregulated	AA-enriched	yes
hsa-miR-542-5p	hsa-miR-542-5p	-1.7648832	downregulated	AA-depleted	yes
hsa-miR-572	hsa-miR-572	-1.7938243	downregulated	AA-depleted	yes
hsa-miR-378*	hsa-miR-378a-5p	-1.4021478	downregulated	AA-depleted	yes

1. Wang, B.D., et al., *Identification and Functional Validation of Reciprocal microRNA-mRNA Pairings in African American Prostate Cancer Disparities.* Clin Cancer Res, 2015. **21**(21): p. 4970-84.

Table S2. Primer sequences for qPCR reactions to examine miRNA and mRNA expression levels. Note that the reverse primer for qPCR assays of miRNAs was the universal primer purchased from Qiagen (Germantown, MD).

**Mature miRNA ID**	**Primer sequences (forward, 5' to 3')**
hsa-miR-34a-5p	TGGCAGTGTCTTAGCTGGTTGT
hsa-miR-96-5p	TTTGGCACTAGCACATTTTTGCT
hsa-miR-99b-5p	CACCCGTAGAACCGACCTTGCG
has-103a-3p	AGCAGCATTGTACAGGGCTATGA

**Gene symbol**	**Sequences of forward primer (5' to 3')**	**Sequences of reverse primer (5' to 3')**
PIK3CB	CATGTCAGGGCTGGTCTTTT	GCACTTTTCCAGCTTTCCTG
HIF1A	CCCAATGGATGATGACTTCC	TGGGTAGGAGATGGAGATGC
IGFBP2	CCCTCAAGTCGGGTATGAAG	ACCTGGTCCAGTTCCTGTTG
MTOR	CCTCACAAGACATCGCTGAA	TCCGGCTGCTGTAGCTTATT
MAPKAPK2	AGAAGTGCTGGGTCCAGAGA	AATTCATACTGGCCCATTCG
EIF1AX	GTACTGGAGAGGGGAGAGCA	TGAAGCTGAGACAAGCAGGA

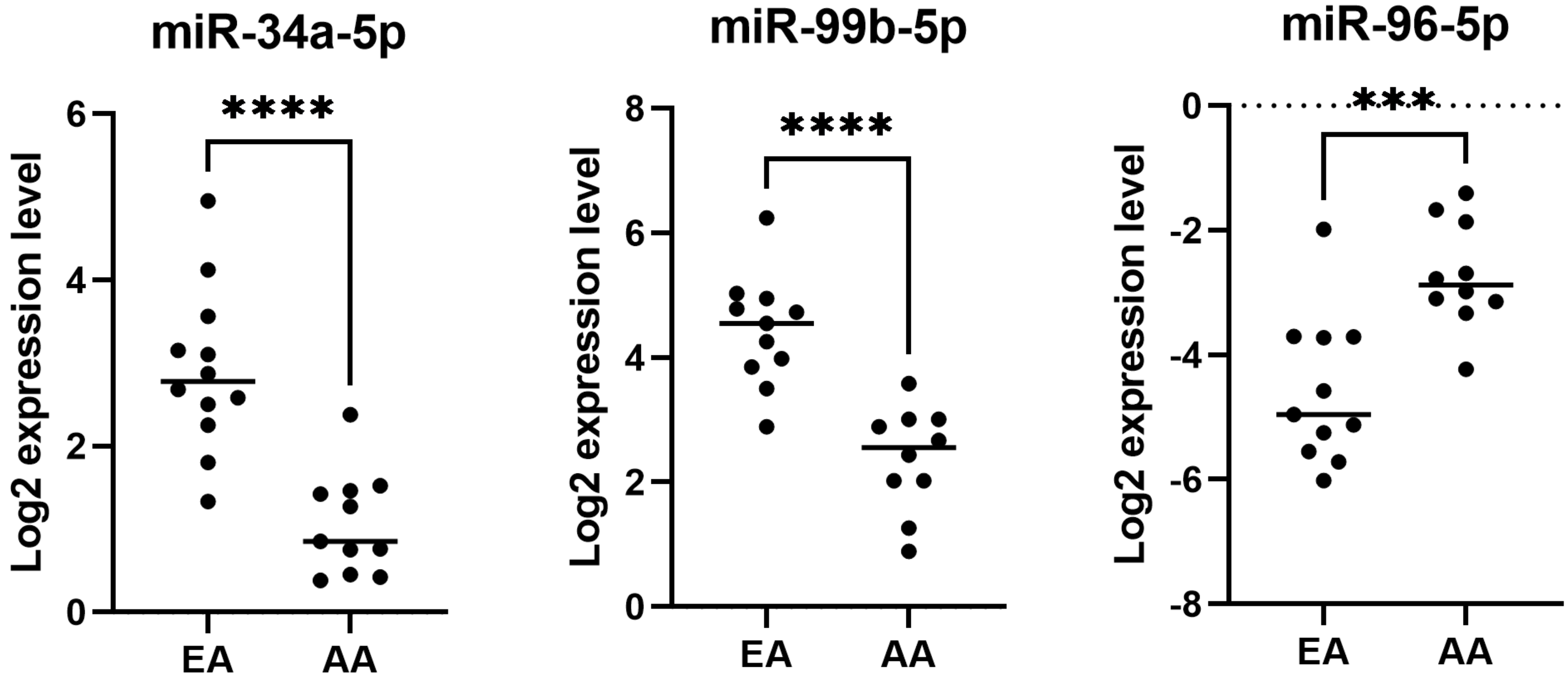

Figure S1. RT-qPCR validation of miR-34a-5p, miR-99b-5p, and miR-96-5p in AA and EA PCa specimens. Scatter dot plots with median values of log2 values for miRNA expression levels from EA and AA PCa were shown. Each dot represented the normalized miRNA expression level from an individual PCa specimen. The relative miRNA expression levels were determined using endogenous miR-103a-3p for normalization. Significance (***p < 0.001, and ****p < 0.0001 in AA PCa vs. EA PCa) was determined based on student t-test.

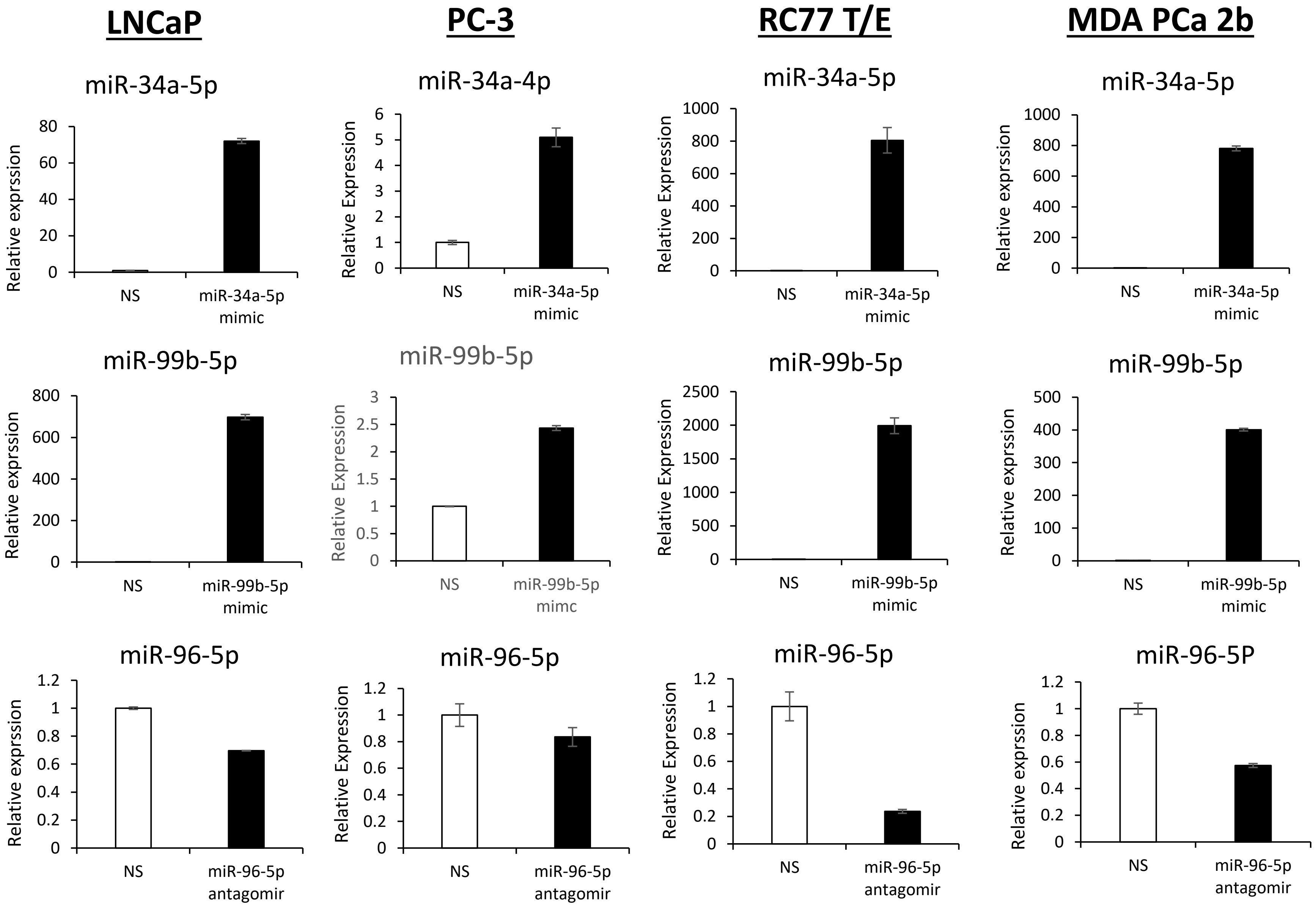

Figure S2. Transfection efficiencies of miR-34a-5p, miR-99b-5p, and miR-96-5p in EA and AA PCa cell lines. RT-qPCR assays revealed comparable transfection efficiencies of miR-34a-5p, miR-99b-5p, and miR-96-5p in LNCaP, PC-3, RC77 T/E, and MDA PCa 2b cells. The smaller fold changes between miR-96-5p and NS transfection in LNCaP and PC-3 simply reflect the fact that only very low levels (close to baseline) of miR-96-5p were detected in LNCaP and PC-3 before miR-96 transfection (as shown in Figure 3A).
